# Frequency of Peripheral Nerve Injury in Trauma in Emergency Settings

**DOI:** 10.7759/cureus.14195

**Published:** 2021-03-30

**Authors:** Saima Mushtaq, Shehla Hina, Humayun Maqbool, Anam Ahmed, Momal Nazim, Erma Hussain, Raja Muhammad Mussab, Besham Kumar

**Affiliations:** 1 Emergency Medicine, Jinnah Post Graduate Medical Centre, Karachi, PAK; 2 Internal Medicine, Akhtar Trust Teaching Hospital, Lahore, PAK; 3 Otolaryngology, Jinnah Post Graduate Medical Centre, Karachi, PAK; 4 Internal Medicine, Abbasi Shaheed Hospital, Karachi, PAK; 5 Surgery, Jinnah Postgraduate Medical Centre, Karachi, PAK; 6 Internal Medicine, Jinnah Postgraduate Medical Centre, Karachi, PAK

**Keywords:** trauma, neurological injuries, road traffic accident

## Abstract

Introduction

Neurological injuries because of trauma and accidents are common but rarely reported or examined in Pakistan. In this study, we will determine the frequency of neurological deficits reported in the emergency unit in patients presenting with acute trauma.

Material

This study was conducted in an emergency unit of tertiary care setting in Karachi, Pakistan. One hundred patients presenting with mild to moderate trauma were enrolled in the study after informed consent. Patients with severe trauma requiring emergency intervention were excluded from the study.

Results

Out of the 100 patients enrolled in our study, 57% presented with neurological deficits after a road traffic accident (RTA), making RTA the most common cause of trauma. The most prominent site of injury was the lower limb (57%). Upper limb examination revealed that out of the 35% patients presenting with upper limb injuries, ten patients (28.6%) had a decreased biceps reflex, while six patients (10.5%) had tingling in their hands. Out of the 57% of patients presenting with lower limb injuries, ten patients (17.5%) had decreased ankle reflexes and six patients (10.5%) had tingling in their legs.

Conclusion

Neurological deficit is very common in patients presenting to emergency settings in Pakistan. Neurologists should be present in emergency centers to perform detail neurological examinations of patients coming to emergency centers, and follow-up visits should be arranged in Neurology clinics for patients suffering from any neurological deficits.

## Introduction

Traumatic neurological injuries are brain or spinal injuries because of mechanical insult to the head from the external physical force [1]. It is a silent epidemic, with an enormous socio-economic burden on the healthcare setting. In the United States of America (USA), it accounts for around 1.4 million emergency room (ER) visits, 275,000 hospital admissions, and 52,000 deaths [1]. Recent research shows an estimated burden of around sixty-nine million individuals suffering traumatic brain injury (TBI) each year, with South-East Asian and Western Pacific regions to be on the top. Lower to middle-income countries have more reported cases of head injury secondary to road traffic accidents (RTAs) and experience three times more TBI than in Higher-income and developed countries with more male prevalence than female. Other leading causes are assault, fall, and estimate-related concussion [2,3]. Neurological injuries because of trauma can have a broad spectrum of presentation including the altered level of consciousness, confusion, vomiting, seizure, coma, focal motor or sensory deficit, and is a major risk factor for post-traumatic epilepsy [4]. Many classifications have been formed for assessing the severity of TBI, with Glasgow Coma Scale; the most widely used one, based on patient’s response to eye-opening, verbal function, and motor function to stimulus [4].

While neurological injuries associated with the spinal cord are catered to, peripheral nerve injuries are often overlooked. This might be the reason the reported incidence of peripheral nerve injury is low (1.5% to 2.8%) [5]. However, it is important to look for peripheral nerve injury after trauma as there is significant associated morbidity particularly when the diagnosis and treatment are delayed [5]. Patients with peripheral nerve injury suffer from various symptoms including muscle weakness, loss of sensation, decreased reflexes, and development of neurological ulcers [6]. Another frequently overlooked nerve injury is facial injury. Facial nerve injury is reported in 7% to 10% reported cases of temporal bone fracture. The most common cause of temporal bone fracture is RTAs, followed by fall and assault [7, 8].

In this study, we will determine the frequency of neurological deficits due to peripheral nerve injury and facial nerve injury reported in the emergency unit in patients presenting with acute trauma.

## Materials and methods

This study was conducted in the Emergency Unit of a tertiary care hospital in Karachi, Pakistan. The duration of this study was from November 2020 to February 2021. One hundred patients presenting with mild to moderate trauma were enrolled in the study after informed consent. Patients with severe trauma requiring emergency intervention were excluded from the study. Patients with spinal injury were also excluded from the study. Patient’s age, gender, the most prominent site of injury, mode of injury, and time since trauma was noted in a self-structured questionnaire. In patients with multiple prominent sites of injury, each site was recorded. The patient’s upper limb, lower limb, and facial neurological examinations were done based on their most prominent site of injury, and findings were recorded in the self-structured questionnaire.

In upper limb examination, patient bicep and wrist reflex were checked. Patient was asked for any paresthesia in fingers and the entire upper limb was examined for loss of sensation. In lower limb examination, patient ankle and patellar reflex were checked Patient was asked for any paresthesia in toes and entire lower limb was examined for loss of sensation. For facial examination, patient was asked to frown to look for facial palsy, smile to look for buccinator paralysis, close eyelid to look for orbicularis oculi paralysis and taste sugar to test for paralysis or injury to facial nerve, glossopharyngeal nerve and vagus nerve

Statistical analysis was done using the Statistical Packages of Social Sciences (SPSS) version. 23.0 (IBM Corporation, Armonk, NY). Data were tabulated as frequency and percentages.

## Results

In this study, the most prominent gender was male (81%). Out of the total number of participants, 55 reached the emergency room within 90 minutes. The most common cause of trauma was a RTA (57%). The most prominent site of injury was the lower limb (57%) (Table 1).

**Table 1:** Characteristics of participants. SD: standard deviation.

Characteristics	Number
Mean ± SD age (in years)	34 ± 11
Gender	
Male	81%
Female	19%
The time between trauma and arrival in emergency room	
Less than 90 minutes	55%
More than 90 minutes	45%
Cause of trauma	
Road traffic accident	57%
Fall	41%
Gunshot	1%
Others	1%
Most prominent site of injury	
Head	15%
Upper Limb	35%
Lower Limb	57%

A total of 35% of patients suffered upper limb injuries. A thorough upper limb examination was performed in these patients which revealed that ten (28.6%) of them had decreased biceps reflexes, while paresthesia in fingers was present in six patients (10.5%) (Table 2).

**Table 2:** Upper limb examination.

Upper limb examination (n=35)	Number (percentage)
Paresthesia in fingers	6 (17.1%)
Bicep reflex decreased (C5 root)	10 (28.6%)
Touch sensation decreased (C6-C8 roots)	9 (25.7%)
Brachioradialis reflex decreased (C6 root)	9 (25.7%)

Out of the 57% of patients presenting with lower limb injuries, a detailed lower limb examination revealed that ten patients (17.5%) had decreased ankle reflexes while six patients (10.5%) complained of a paresthesia in toes (Table 3).

**Table 3:** Lower limb examination.

Lower limb examination (n=57)	Number (percentage)
Paresthesia in toes	6 (10.5%)
Ankle reflex decreased (S1 roots)	10 (17.5%)
Touch sensation decreased (L3-S2 roots)	6 (10.3%)
Patellar reflex decreased (L4 roots)	12 (20.7%)

In the facial examination, sign of facial palsy in the forehead (asymmetrical wrinkles) was present in four (26.7%) participants. Five patients (33.3%) could not keep their eyelids forcibly closed due to weakness of orbicularis oculis (Table 4).

**Table 4:** Facial examination.

Facial examination (n=15)	Number (percentage)
Asymmetrical wrinkle forehead (facial palsy)	4 (26.7%)
Cannot keep eyelid close forcibly (orbicularis oculi weakness)	5 (33.3%)
Cannot show his teeth/smile in an equal manner (facial palsy)	3 (20%)
Cannot blow cheeks (Buccinator muscle weakness)	4 (26.7%)
No taste (injury to facial nerve, glossopharyngeal nerve or vagus nerve)	3 (21.4%)

## Discussion

This study clearly outlines how common is the occurrence of neurological deficits in patients presenting with acute trauma in Karachi, Pakistan. Nearly one-fourth of the patients with upper limb injuries had all upper limb reflexes decreased. In patients with predominantly lower limb injuries, nearly one-fifth had decreased patellar and ankle reflexes.

We found that a full neurological examination is mostly carried out only in patients with a suspected spinal cord injury. In patients who are not suspected of having any spinal cord injury, a full neurological examination is routinely not undertaken due to either a lack or absence of characteristic symptoms or when the symptoms are present but considered 'mild'. [9,10]. However, Fujimura Y et al., in their study stated that even a mild neurological deficit at the time of trauma might indicate unstable injury [11].

Neurological deficit in trauma settings in participants without spinal trauma is due to peripheral nerve injury. The most common reason for peripheral nerve injury is fractures of adjacent bones [12]. Usually, multiple nerves are involved in injury [13]. In our study, the neurological deficit was more common in the upper limb compared to the lower limb. Various other studies have reported a similar finding. [14,15]. This higher involvement of nerves in the forearm, wrist, and hand is due to their superficial location and the exposure of these regions in daily work with sharp cutting objects [13].

It is important to determine the exact cause of trauma, as it has prognostic value while treating neurological deficit. The outcome of neurological deficit is better in patients with penetrating injuries compared to blunt trauma [16]. In peripheral nerve injury where vascular trauma is also present is very critical, as the combination of both of them can lead to permanent limb dysfunction [16,17].

Facial neurological deficits were found to be present in one-fourth. Facial nerve injury can be due to blunt or penetrating trauma, and may result in facial paralysis. Therefore, the initial evaluation must include a full motor and sensory examination. Furthermore, investigations like High-Resolution Computed Tomography (HRCT) should be used for localization of nerve injury in high-risk cases, such as in suspected cases of temporal bone injury [18].

To the best of our knowledge, this study is the first of its kind, which explores the frequency of peripheral nerve injury and facial nerve injury in Pakistan. However, the study has its limitation as well. First, since it was a cross-sectional study, the long-term impact of nerve injury was not evaluated. Second, since the study was done in a single center, the sample size was not diverse.

Based on this study, we determine that neurological injuries are frequent in the setting of trauma. Because of the increased load of patients in emergency settings in tertiary care setting of developing countries, often complete systemic examination of patient cannot be performed. It is important that a system should be in place to ensure any patient presenting with undergoes complete physical and systemic examination, in addition to being treated for his/her injuries. This will allow early diagnosis of neurological injuries associated with trauma and lead to early recovery as well.

## Conclusions

In our study, peripheral nerve injuries were common in both upper and lower limb, as demonstrated by decrease in reflex, sensation and paresthesia. Facial nerve injury as indicated by weakness of buccinator muscles, orbicularis orbis and signs of facial palsy were also commonly presented. In a country like Pakistan, where resources are minimal, the neurological examination is rarely performed in an emergency setting. Even when performed, there is no follow-up of patients to see if the neurological deficit has resolved. Therefore, we believe that Neurologists should be present as routine in emergency care settings to perform the relevant examinations of trauma patients, and follow-up visits should always be arranged for patients with positive findings of neurological deficits.
